# Contrasting Roles for TLR Ligands in HIV-1 Pathogenesis

**DOI:** 10.1371/journal.pone.0012831

**Published:** 2010-09-20

**Authors:** Beda Brichacek, Christophe Vanpouille, Yana Kiselyeva, Angelique Biancotto, Melanie Merbah, Ivan Hirsch, Andrea Lisco, Jean Charles Grivel, Leonid Margolis

**Affiliations:** 1 Section of Intercellular Interactions, Program in Physical Biology, National Institute of Child Health and Human Development, National Institutes of Health, Bethesda, Maryland, United States of America; 2 Centre de Recherche en Cancérologie de Marseille, UMR 891 INSERM, Institut Paoli-Calmettes, Universite de la Mediterrane, Marseille, France; University of Toronto, Canada

## Abstract

The first line of a host's response to various pathogens is triggered by their engagement of cellular pattern recognition receptors (PRRs). Binding of microbial ligands to these receptors leads to the induction of a variety of cellular factors that alter intracellular and extracellular environment and interfere directly or indirectly with the life cycle of the triggering pathogen. Such changes may also affect any coinfecting microbe. Using ligands to Toll-like receptors (TLRs) 5 and 9, we examined their effect on human immunodeficiency virus (HIV)-1 replication in lymphoid tissue *ex vivo*. We found marked differences in the outcomes of such treatment. While flagellin (TLR5 agonist) treatment enhanced replication of CC chemokine receptor 5 (CCR 5)-tropic and CXC chemokine receptor 4 (CXCR4)-tropic HIV-1, treatment with oligodeoxynucleotide (ODN) M362 (TLR9 agonist) suppressed both viral variants. The differential effects of these TLR ligands on HIV-1 replication correlated with changes in production of CC chemokines CCL3, CCL4, CCL5, and of CXC chemokines CXCL10, and CXCL12 in the ligand-treated HIV-1-infected tissues. The nature and/or magnitude of these changes were dependent on the ligand as well as on the HIV-1 viral strain. Moreover, the tested ligands differed in their ability to induce cellular activation as evaluated by the expression of the cluster of differentiation markers (CD) 25, CD38, CD39, CD69, CD154, and human leukocyte antigen D related (HLA)-DR as well as of a cell proliferation marker, Ki67, and of CCR5. No significant effect of the ligand treatment was observed on apoptosis and cell death/loss in the treated lymphoid tissue *ex vivo*. Our results suggest that binding of microbial ligands to TLRs is one of the mechanisms that mediate interactions between coinfected microbes and HIV-1 in human tissues. Thus, the engagement of appropriate TLRs by microbial molecules or their mimetic might become a new strategy for HIV therapy or prevention.

## Introduction

HIV-1 infection, as well as the progression of HIV-1 disease, usually occurs in the presence of other microbes [1]–[5]. These infectious agents typically serve as copathogens, facilitating HIV-1 transmission and aggravating the clinical course of HIV-1 disease. However, some infectious agents seem to have an opposite effect and can alleviate the course of HIV-1 disease [6]–[10]. Evidence exists that these microbes interact locally with HIV-1 by up- or down-regulating HIV-1 coreceptors [11]–[13] and receptors [14] as well as by inducing changes in the production of various cytokines [11], [13]. While the mechanisms by which microbes interact with HIV-1 are not fully understood, it is clear that their invasion into the human body triggers a cascade of events that can affect HIV-1 pathogenesis.

One of the first events in the interaction of the human body with an invading microorganism is the engagement of cellular PRRs (for review see [15]–[17]). The best-characterized class of PRRs are the Toll-like receptors (TLRs), a family of pathogen sensors that trigger local inflammation, recruitment of effector cells, and secretion of cytokines that modulate both the innate and adaptive immune responses [18]–[20]. TLRs are expressed in a wide variety of cells, located in different tissues and organs including lymphoid organs [20]–[25]. Activation of TLRs engages multiple intracellular adaptor and signaling proteins and triggers several cascades of innate immune response [17]. Since the pattern of expression of TLRs depends on a particular cell subtype and on the engagement of a ligand to a particular TLR receptor, the system responds to different pathogens differently [17], [26]. In general, this response may include cell activation and differentiation [27], [28], secretion of type I IFNs, and/or production of proinflamatory cytokines [17], [29]. These responses to TLRs engagement together with the response to the engagement of other PRRs and various cellular factors also regulate adaptive immunity [16]. Because of the profound role of TLRs engagement in immune response, TLR agonists, antagonists, specific antibodies or shRNA are now being tested as novel therapeutics and adjuvants for vaccines [26], [30]–[40].

As first responders to an invading virus, TLRs and other PRRs play a critical role in directing the anti-viral host responses [41]–[46]. These responses can be affected by simultaneous signaling from PRRs engaged by other coinfecting microbes. These effects have been studied by applying TLR ligands to HIV-1-infected cell lines, isolated primary cells, human lymphocyte aggregate cultures and experimental animals [47]–[53].

However, critical events in HIV-1 pathogenesis occur in the context of structured human lymphoid organs [54]–[57]. Therefore, here we describe the effects of TLR engagement on HIV-1 replication in lymphoid tissue using explants of palatine tonsils, where TLRs are broadly expressed [24], [25]. This system was developed in our laboratory and has been used to address HIV-1 tissue pathogenesis as well as HIV-1 interactions with various microbes [12], [14], [58]–[60]. We report that in human lymphoid tissue, different TLR ligands modulate HIV replication both positively and negatively and that a TLR-triggered cell activation and change of cytokine spectra are involved in these modulations.

## Results

### TLR ligands modulate HIV-1 replication in human lymphoid tissue *ex vivo*

**Figure 1:**
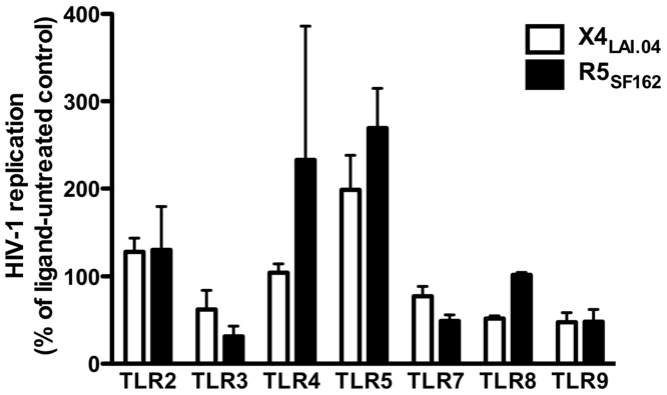
Effect of TLR ligands on HIV-1 replication in human lymphoid tissue *ex vivo*. Blocks of human lymphoid tissue were cultured overnight in medium alone or pretreated with ligands for TLR2 (LPS from Porphyromonas gingivalis), TLR3 (poly (I:C)), TLR4 (LPS from E. coli K12), TLR5 (flagellin from S. typhimurium), TLR7 (loxoribine), TLR8 (ssRNA40/LyoVec), and TLR9 (CpG oligonucleotide type C: ODN M362). Following pretreatment, tissues were infected with X4 or R5 HIV-1 (X4_LAI.04_ or R5_SF162_). HIV-1 replication was monitored with p24 ELISA. Cumulative values for HIV-1 p24 release in ligand-treated cultures are expressed as percentages of the values obtained in ligand-untreated cultures. Plotted data represent means (± SEM) of n experiments for X4 or R5, respectively, with p characterizing statistical significance of the difference between untreated and TLR ligand-treated tissues. The ligands to the following TLRs were used: TLR2 (n = 4; p = 0.171 and n = 4; p = 0.582), TLR3 (n = 5; p = 0.160 and n = 5; p = 0.004), TLR4 (n = 4; p = 0.700 and n = 5; p = 0.435), TLR5 (n = 6; p = 0.016 and n = 4; p = 0.034), TLR7 (n = 5; p = 0.115 and n = 6; p = 0.001), TLR8 (n = 2; p = 0.043 and n = 3; p = 0.632), TLR9 (n = 5; p = 0.008 and n = 5; p = 0.021).

Blocks of human lymphoid tissue were pretreated overnight with ligands for TLR2 (LPS from *Porphyromonas gingivalis*), TLR3 (poly (I:C)), TLR4 (LPS from *E. coli* K12), TLR5 (flagellin from *S. typhimurium*), TLR7 (Loxoribine), TLR8 (ssRNA40/LyoVec), and TLR9 (CpG oligonucleotide type C: ODN M362). The pretreated tissues were infected with R5 or X4 HIV-1 (X4_LAI.04_ or R5_SF162_). Viral replication was evaluated from tissues' release of p24 into the culture medium over 15 days of infection. Results of these experiments are shown in Figure 1. TLR ligands had diverse effects on HIV-1 replication ranging from suppression of both R5 and X4 HIV-1 variants, through differential effects on R5 and X4 HIV-1 variants, to upregulation of both R5 and X4 HIV-1. To investigate the mechanisms of modulation of HIV-1 replication by TLR ligands, we further focused on two ligands that affect HIV in opposite ways: flagellin and ODN M362. The TLR5 ligand flagellin enhanced replication of both R5_SF162_ and X4_LAI.04_ HIV-1 (*n* = 4, *p* = 0.034 and *n* = 6, *p* = 0.016, for R5 and X4 HIV-1, respectively), whereas TLR9 ligand ODN M362 suppressed both HIV-1 variants (*n* = 5, *p* = 0.021 and *n* = 5, *p* = 0.008, for R5 and X4, respectively) (Fig. 1).

### TLR9 ligand ODN M362 suppresses HIV-1 replication in tonsillar tissue *ex vivo*

**Figure 2:**
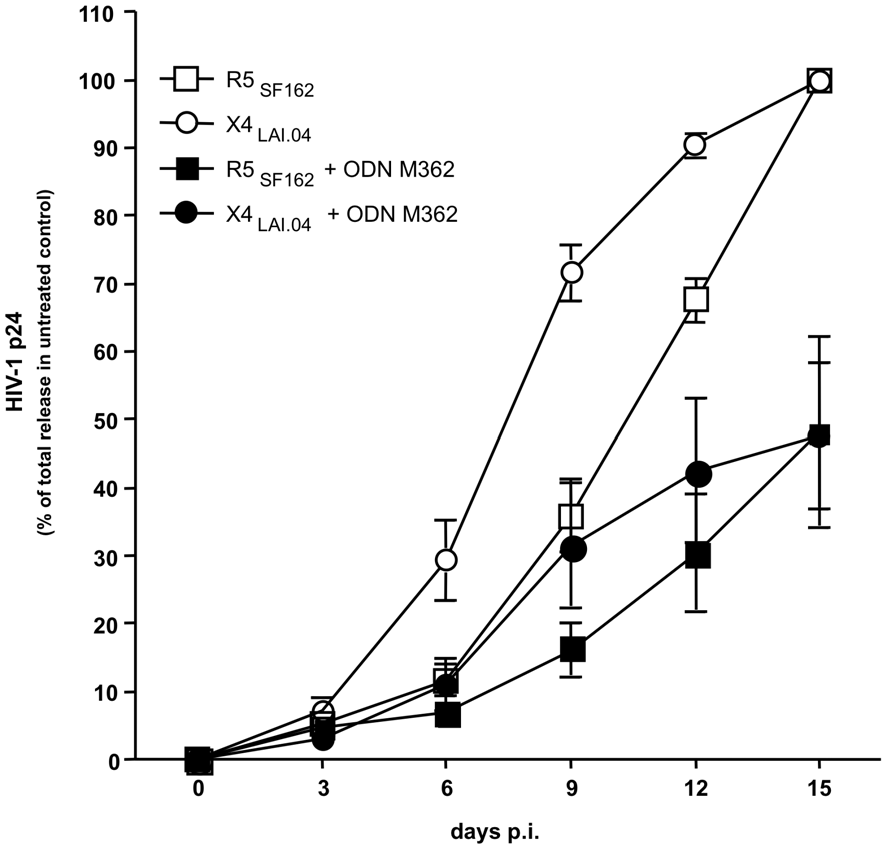
Suppression of HIV-1 replication in human lymphoid tissue *ex vivo* treated with the TLR9 ligand ODN M362. Release of HIV-1 was monitored in the culture medium of ODN M362-treated and -untreated cultures. Each datum point reflects cumulative p24 release. Data were normalized to the total release of p24 in untreated cultures. Presented data are means (± SEM) of four experiments. Differences between ODN M362-treated and -untreated cultures are statistically significant (p<0.05) at days 6 to 15 for X4_LAI.04_ infection and at days 9 to 15 for R5_SF162_ infection.

Treatment of HIV-1 in *ex vivo*-infected cultures with ODN M362 decreased HIV production (Fig. 2). In the treated tissues, replication of X4_LAI.04_ became typically detectable 6 days post infection (p.i.), while in donor-matched tissues not treated with ODN M362 it was typically detectable at day 3 p.i. A similar 3-day delay was observed for R5_SF162_ replication in tissues treated with ODN M362. In ODN M362-treated HIV-1-infected tissues, the maximum p24 release was on average 3.4-fold lower (range 1.1 to 9.5; *n* = 5) for X4_LAI.04_ and 3.0-fold lower (range 1.1 to 7.8; *n* = 5) for R5_SF162_ compared with donor-matched HIV-1-infected tissues not treated with ODN M362. Consequently, in tissues treated with ODN362 the total p24 production over 15 days of infection was significantly decreased to the level of 47.7%±8.3% (*p*<0.02, *n* = 5) for X4_LAI.04_ and to the level of 48.1%±13.0% (*p*<0.01, *n* = 5) for R5_SF162_ relative to matched infected tissues not treated with this ligand (Fig. 2).

To obtain further evidence that the HIV-1 suppression by ODN M362 is mediated by the TLR9 pathway we tried to prevent this suppression by TLR9 antagonists. We treated the tissue simultaneously with TLR9 ligand ODN M362 and a 5-fold excess of TLR9 antagonist (TTAGGG)4. The total R5 HIV-1 replication over 18 days in the presence of both compounds (added repeatedly with every medium change) was 54.7%±8.3 (mean ± SEM) (p∼0.01, *n* = 5) higher than in matched tissue blocks treated with the agonist only, although the level of replication was not restored to the level of the untreated control.

### Flagellin enhances HIV-1 replication in tonsillar tissue *ex vivo*

**Figure 3:**
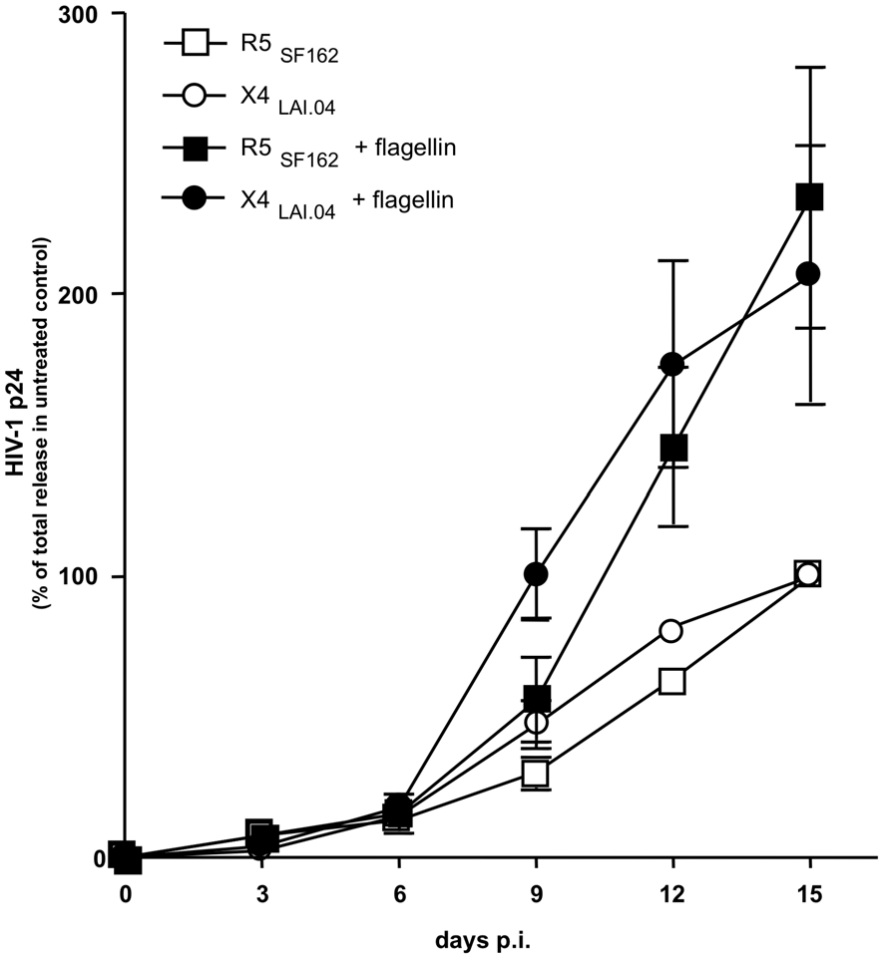
Enhancement of HIV-1 replication in human lymphoid tissue *ex vivo* treated with the TLR5 ligand flagellin. Release of HIV-1 was monitored in the culture medium of flagellin-treated and -untreated cultures. Each datum point reflects cumulative p24 release. Data were normalized to the total release of p24 in untreated cultures over the period of 15 days. Presented data are means (± SEM) of four experiments. Differences between flagellin-treated and -untreated cultures are statistically significant (p<0.05) at days 9 to 15 for X4_LAI.04_ infection and at days 12 to 15 for R5_SF162_ infection.

HIV-1 p24 release was increased in tissues treated with flagellin from *S. typhimurium* compared with matched HIV-1-infected tissues not treated with this TLR5 ligand (Fig. 3). Replication of both X4_LAI.04_ and R5_SF162_ typically became detectable in the treated and untreated tissues simultaneously (day 3 p.i.); the maximum production of p24 was increased by flagellin 2.4-fold (range 1.2 to 3.5, median 2.4, *n* = 5) for X4_LAI.04_ and 3.2-fold (range 1.5 to 4.3, median 3.4, *n* = 5) for R5_SF162_ compared with matched untreated tissue. On average, the cumulative p24 release by flagellin-treated tissues reached 198.5%±39.5% (*p*<0.05, *n* = 5) and 269.4%±45.5% (*p*<0.05, *n* = 5) for X4_LAI.04_ and R5_SF162_, respectively, relative to p24 release by infected tissues not treated with this ligand (Fig. 3).

### Effects of ODN M362 and flagellin on chemokine production in HIV-1 infected tonsillar tissue *ex vivo*

**Figure 4:**
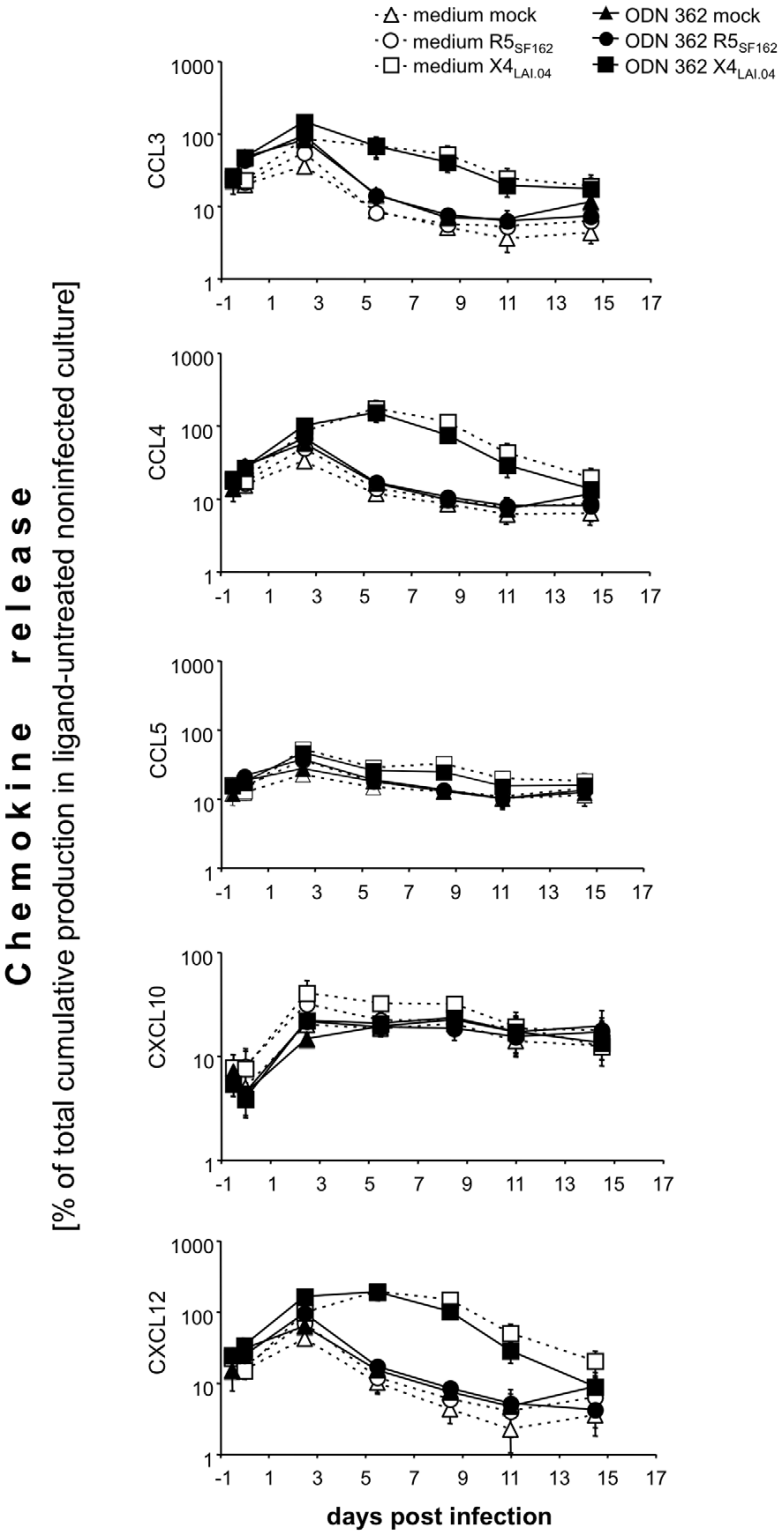
Effects of ODN M362 on chemokines production in HIV-1-infected lymphoid tissue *ex vivo*. Blocks of tonsillar tissue were treated with ODN M362 and subsequently infected with HIV-1 X4_LAI.04_ or R5_SF162_. Release of CCL3, CCL4, CCL5, CXCL10, and CXCL12 was monitored in the culture medium with the multiplexed fluorescent microsphere immunoassay. Concentrations of released chemokines at individual time points were normalized to their total cumulative release in matched untreated cultures. Presented data are means of these normalized values (± SEM) from 4 to 7 experiments. Statistical analysis revealed significant difference between: (i) uninfected and X4_LAI.04_ –infected (but not R5-infected) tissues in the release of CCL3, CCL4, CCL5, CXCL10, and CXCL12 (*p*<0.046, *n* = 5 to 7); (ii) uninfected mock- and ODN M362-treated tissues in production of CCL3, CCL4, CCL5, and CXCL12 (*p*<0.031, *n* = 5); (iii) R5_SF162_-infected tissues and these tissues treated with ODN M362 in the production of CCL3, CCL4, CCL5, and CXCL12 (*p*<0.04; *n* = 6); (iv) X4_LAI.04_- infected tissues and these tissues treated with ODN M362, in production of CCL4 and CXCL12 (*p*<0.046, *n* = 6).

**Figure 5:**
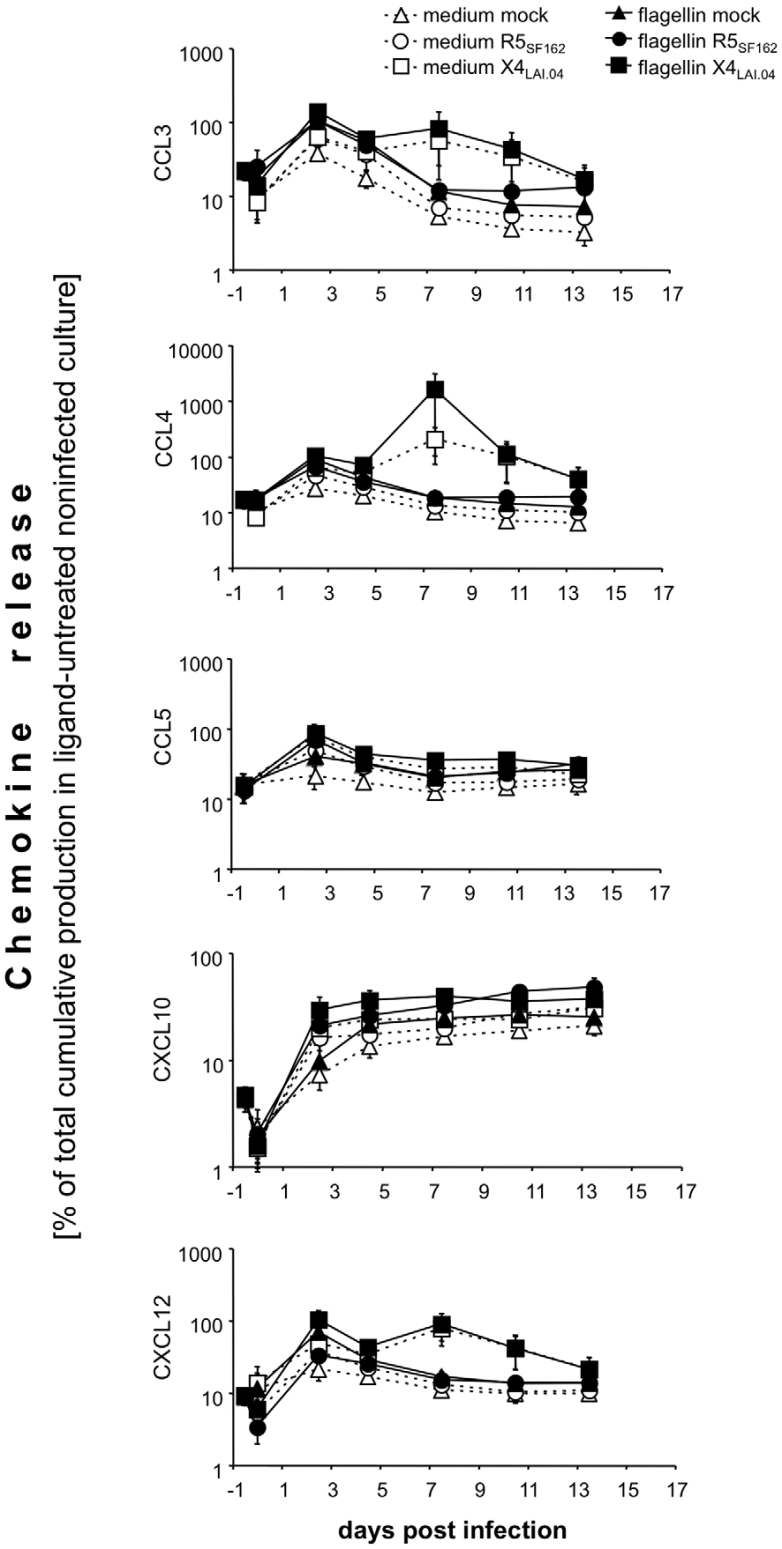
Effects of flagellin on chemokines production in HIV-1-infected lymphoid tissue *ex vivo*. Blocks of tonsillar tissue were treated with flagellin and subsequently infected with HIV-1 X4_LAI.04_ or R5_SF162_. Release of CCL3, CCL4, CCL5, CXCL10, and CXCL12 was monitored in the culture medium with the multiplexed fluorescent microsphere immunoassay. Concentrations of released chemokines at individual time points were normalized to their total cumulative release in matched untreated cultures. Presented data are means of these normalized values (± SEM) from 4 to 7 experiments. Statistical analysis (see the Results) revealed significant difference between (i) uninfected and X4_LAI.04_ –infected (but not R5-infected) tissues in the release of CCL3, CCL4, CCL5, and CXCL10 (*p*<0.046, *n* = 5 to 7); (ii) R5_SF162_-infected tissues and these tissues treated with flagellin in the release of CCL3, CCL4, CCL5, CXCL12, and CXCL10 (*p*<0.016, *n* = 6); (ii) X4_LAI.04_ -infected tissues and these tissues treated with flagellin in the release of CCL3, CCL4, and CXCL10 (*p*<0.046, *n* = 6).

To investigate whether the effects of the TLR ligands ODN M362 and flagellin on HIV-1 replication are mediated by chemokines, we compared the release of relevant chemokines in donor-matched tissues treated with TLR ligands and/or infected by HIV-1, and in untreated tissues. We measured CCL3, CCL4, CCL5, and CXCL12, all shown to be associated with inhibition of HIV-1 entry, and CXCL10, a major chemoattractant of activated T cells and monocytes [61]–[63]. Below, we describe the effect of (i) the TLR ligands ODN M362 and flagellin, (ii) HIV-1 R5 and X4, and (iii) a combination of TLR ligands and HIV-1 on these chemokines. The results of these experiments are presented in Figures 4 and 5.

- Treatment with either ODN M362 or flagellin induced a transient increase in production of CCL3, CCL4, CCL5, and CXCL12 with a peak within 3 days post-treatment. At this time, the average levels of CCL3, CCL4, CCL5, and CXCL12 in ODN M362-treated tissues were 1.3- to 2.1-fold higher than those in untreated tissues (*p*<0.031, *n* = 5) (Fig. 4). Although a similar increase was observed in flagellin-treated tissues, it did not reach statistical significance (*p*>0.063, *n* = 4) (Fig. 5). Neither ligand induced a statistically significant change in the release of CXCL10 (*p*>0.13).
- As was shown earlier [60] and confirmed here (Figs. 4 & 5), R5 HIV-1 replication in tonsillar tissue *ex vivo* did not affect the production of tested CC-chemokines (*p*>0.063, *n* = 7). The release of CXCL10 and CXCL12 from R5_SF162_-infected tonsillar tissues into the culture medium was not significantly changed either (*p*>0.19). In contrast, in X4_LAI.04_-infected tissues production of CCL3, CCL4, CCL5, CXCL10, and CXCL12 was significantly increased compared with that in uninfected tissues and reached a maximum 5 to 9 days post-infection. At their peak, the concentrations of CCL3, CCL4, CCL5, CXCL10, and CXCL12 released by X4_LAI.04_-infected tissues were approximately 20, 20, 2, 1.8, and 80 times higher, respectively than those in uninfected cultures at the same time (*p*<0.008, <0.008, <0.008, <0.039, and <0.046, respectively, *n* = 7). In Figures 4 and 5, we present these data normalized by the cumulative production of corresponding cytokines in untreated uninfected donor-matched tissues to account for the variability of tissue obtained from different donors. The kinetics of secretion of these cytokines mirrored the production of X4 HIV-1_LAI.04_ p24 by the infected tonsillar tissues but preceded it by 3 days (*r* = 0.9, *p*<0.0001).
- In R5_SF162_-infected tissues treated with ODN M362, there were significant increases in the concentrations of CCL3, CCL4, CCL5, and CXCL12 (by 90%, 60%, 40%, and 40%, respectively; *p*<0.03, <0.015, <0.04, <0.015, *n* = 6). These increases were detectable as early as 3 days post-infection and waned later in the course of infection. In contrast, the amount of CXCL10 released from R5_SF162_-infected tissues pretreated with ODN M362 decreased by 30% (*p*<0.0001) relative to that released from R5_SF162_-infected tissue not treated with the ligand.

In X4_LAI.04_ -infected tissues treated with ODN M362, there was an increased release of CCL4 and CXCL12 into the culture medium compared with ligand-untreated X4_LAI.04_-infected tissues. At 60 h post-infection, the respective concentrations of these chemokines were 30% and 70% higher (*p*<0.04, *n* = 6). As the X4_LAI.04_ infection of the ODN M362-treated tissues progressed, secretion of CCL4 and CXCL12 decreased and on day 9 p.i. became 30% lower (*p* = 0.047) than that produced by matched X4_LAI.04_-infected ligand-untreated tissues. In contrast, the concentration of CXCL10 in ODN M362-treated X4_LAI.04_-infected tissues was lower throughout the entire course of infection and reached 74% on day 6 p.i. (*p* = 0.047, *n* = 6).

In R5_SF162_ infected tissues treated with flagellin, there was an increased release of all measured cytokines compared with tissues infected with R5_SF162_ but not treated with this ligand. On day 6 and/or 9 post–R5_SF162_ infection, the concentrations of these cytokines were 15 to 78% higher than those in matched control tissues (*p*<0.016, *n* = 6).

In X4_LAI.04_ infected tissues treated with flagellin, there was a significant increase in the levels of CCL3, CCL4, and CXCL10 (by 130%, 60%, and 30%, respectively; *p*<0.047, *n* = 6) already on day 3 post-infection. On day 9, the levels of CXCL10 were increased by 60% compared with those in X4_LAI.04_-infected tissues not treated with ligands (*p* = 0.016, *n* = 6). Also, X4_LAI.04_ infection of tissues treated with flagellin resulted in an increase of CCL5 secretion. However, this increase reached statistical significance starting on day 8 p.i. (40%; *p* = 0.016). X4_LAI.04_ infection of tissues treated with flagellin did not affect the release of CXCL12.

**Figure 6:**
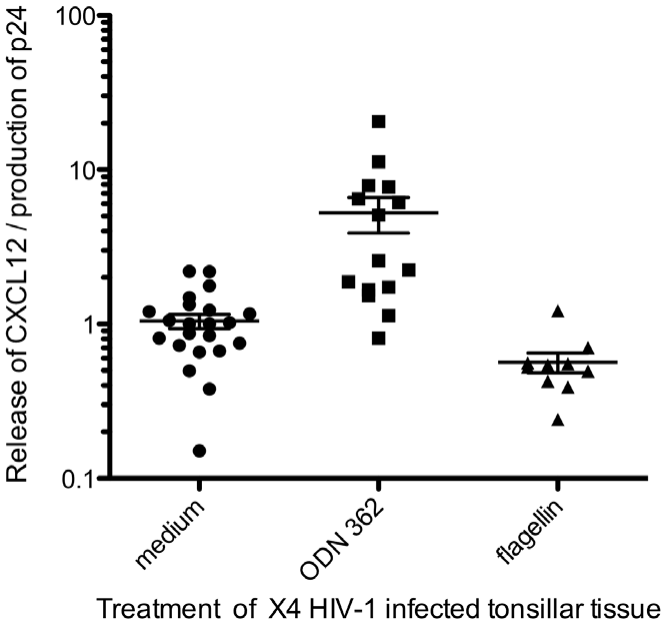
ODN M362 and flagellin modulate CXCL12 release HIV-1 X4_LAI.04_ -infected lymphoid tissue *ex vivo*. Blocks of tonsillar tissue were treated either with flagellin (▴) or with ODN M362 (▪) or mock-treated (•), and subsequently infected with HIV-1 X4_LAI.04_. Concentrations of CXCL12 and p24 were determined in the culture supernatants every 3^rd^ day for 12 days following the infection. Data were normalized to the maximums released in matched X4_LAI.04_-infected ligand-untreated cultures. Each datum point represents a ratio between released CXCL12 and HIV-1 p24 in each of 47 HIV-1 positive supernatants. On average, in ODN M362 treated cultures, the relative release of CXCL12 per unit of HIV-1 p24 was 5 times higher (*p*<0.001) whereas in flagellin-pretreated cultures, it was twice lower (*p* = 0.01) than in ligand-untreated culture.

In contrast, to the release of CXCL12 per culture, the relative release of this cytokine per unit of HIV-1 p24 in ODN M362 treated cultures, was on average, 5 times higher (*p*<0.001) whereas in flagellin-pretreated cultures, it was twice lower (*p* = 0.01) than in ligand-untreated culture (Fig. 6).

### Effects of flagellin and of ODN M362 on HIV-1 coreceptor expression in *ex vivo* tonsillar tissue

We treated tonsillar tissues *ex vivo* with TLR ligands ODN M362 and flagellin to investigate the effects of these compounds on the expression of HIV-1 coreceptors CCR5 and CXCR4 on CD3^+^CD4^+^ T cells. Cells expressing CD3, CD4, CD8, CCR5, and CXCR4 in TLR ligand-treated tonsillar tissues were identified with flow cytometry, and their numbers were compared with those in control untreated tissues. During culture, cells emigrate from the tissue blocks and accumulate within the gelfoam and in the culture medium. Therefore, at least two cellular populations can be distinguished: those residing in the tissue blocks and those which emigrated [64].

In untreated tonsillar blocks, CD3^+^ cells constituted 45%±6.7% (*n* = 3) of lymphocytes. CD4^+^ and CD8^+^ T cells constituted 56.6%±5.9% and 15.5%±6.7%, of CD3^+^ cells, respectively. No statistically significant changes in the fractions of CD3^+^, CD3^+^CD4^+^ or CD3^+^CD8^+^, and CD3^+^CD4^+^CCR5^+^ lymphocytes that resided in tonsillar bocks or emigrated out after ODN M362 or flagellin were detected.

Nearly all CD4 positive T lymphocytes express CXCR4 [65]. None of the TLR ligands induced a decrease in the fraction of CXCR4-positive CD4^+^ T cells or a considerable shift in the level of expression of CXCR4 on their surface as measured by the intensity of the staining.

### Flagellin modulates the expression of activation markers on CD4^+^CD8^−^ T lymphocytes

Since HIV-1 replicates predominantly in activated T cells [66]–[68], we evaluated the effect of flagellin and ODN M362 on the expression of activation markers on CD4^+^ T cells. In particular, we stained tissue cells for CD3, CD4, CD8, CD25, HLA-DR, CD38, CD39, CD69, CD154, as well as for Ki67.

**Figure 7:**
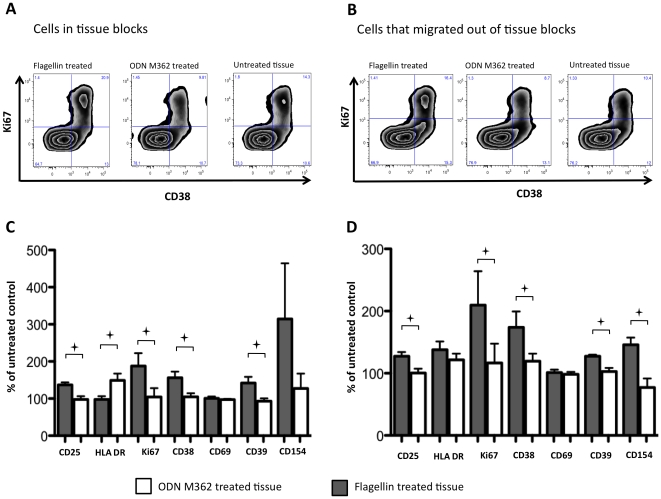
Effect of ODN M362 and flagellin on CD4^+^ T-cell activation. Blocks of tonsillar tissue were treated with either flagellin or ODN M362 for 7 days. Expression of activation markers CD25, HLA-DR, CD38, CD69, CD39, CD154 on CD4^+^CD8^−^ CD3^+^ lymphocytes (Panels A & C) and of cell-proliferation marker Ki67 in these cells (panels B & D) were monitored with flow cytometry in tissue blocks (Panels A & B) and in the fraction of cells emigrated from the tonsillar tissues (Panels C & D). Panels A & B show representative staining of CD3^+^CD4^+^CD8^−^ lymphocytes for CD38 and Ki67. Data were compensated and analyzed with FlowJo version 9 (Treestar, Ashland, OR, USA). The plotted data represent means (± SEM) of fractions of CD4^+^CD8^−^ CD3^+^ lymphocytes positive for particular activation marker from four experiments. Data are normalized to the corresponding fraction of CD4^+^CD8^−^ CD3^+^ lymphocytes from matched untreated control tissue. **^+^** marks statistically significant (p<0.04) difference between flagellin- and ODN M362-treated tissue for a particular activation marker.

Six days after the treatment of tonsillar tissue blocks with ODN M362 or flagellin, the cells were analyzed with flow cytometry. In general, in flagellin-treated tissues the fraction of cells expressing the activation markers was increased (Fig. 7). In cells that were isolated from the tissue blocks, the increases reached statistical significance for CD25 and CD38 (137±7% and 156±17% of the untreated tissue, respectively, *p*<0.05). In cells that migrated from the tissue blocks, the increases reached statistical significance for CD25, CD39 and CD154 (127±7%, 128±2%, 146±12% of the untreated tissue, respectively, *p*<0.03). In contrast, in tissues treated with ODN M362 there were no statistically significant changes in the fraction of CD4 T cells expressing the activation markers, except for HLA-DR which was increased on the CD3^+^CD4^+^CD8^−^ cells that migrated out of the tissue blocks (122±7 of the untreated tissue, *p*<0.002). As the result of these changes, there were statistically significant differences between tissues treated with the two ligands in the fractions of cells expressing CD25, CD38, HLA-DR, CD39, and Ki67 (*p*<0.04). Similarly, there were significant differences in expression of these markers, except for HLA-DR, between the tissues treated with flagellin and those treated with ODN M362 in the fractions of CD4 T cells that emigrated from the tissue blocks (*p*<0.04).

Also, we analyzed the cycling of CD4 T cells in tissues treated with flagellin or ODN M362. As shown in Figure 7, flagellin treatment resulted in almost doubling of the number of Ki67^+^ CD4 T cells remaining in the blocks and which emigrated from them. Thus, in contrast to ODN M362, both the fraction of cycling cells and cells expressing most of the activation markers were increased by flagellin treatment (Fig. 7).

Finally, in flagellin-treated tissues, there were more CD3^+^ cells with higher forward side scatter (1.94%±0.2%) (“lymphoblasts”) than in ODN M362-treated tissue (1.15%±0.3%) (*p* = 0.0132, *n* = 3).

Thus, flagellin leads to higher activation of CD3^+^CD4^+^ lymphocytes in tonsillar tissue than ODN M362.

### Effects of ODN M362 and flagellin on CD4^+^CD8^−^ apoptosis and cell loss in tonsillar tissues *ex vivo*

Neither annexin V staining nor cell counting revealed statistically significant differences between tissues treated with ODN M362 or flagellin and untreated tissues. In untreated tonsillar blocks 6%±2.6% of CD3^+^CD4^+^ lymphocytes bound annexin V, while in ODN M362- and flagellin-treated tissues 4.4%±0.5% and 3.5%±1.1% of CD3^+^CD4^+^ lymphocytes, respectively, bound this apoptotic marker. Although these differences were more pronounced in cells that emigrated from the tissue blocks, they did not reach statistical significance (0.075<*p*<0.59, for all comparisons). The fractions of annexin V-binding CD3^+^CD4^+^ lymphocytes among the emigrated cells were 7.4%±1.9%, 5.1%±0.3%, and 2.9%±0.9% in untreated, ODN M362-treated, and flagellin-treated cultures, respectively. Moreover, no significant differences in cell loss were detected between untreated tissues and tissues treated with either ligand (0.16<*p*<0.77).

## Discussion

Clinical and experimental evidence suggests that various microbes are able to affect the HIV-1 life cycle by changing the systemic and/or local environment [69]–[75] or by interacting with HIV-1 directly [76], [77]. For example, microbial infection may lead to the modulation of cell activation (HSV-2) [78], to changes in receptor expression (HSV-2, HHV-7, GBV-C) [13], [14], [79]–[81], and to the release of chemokines (HHV-6, GBV-C) [11], [13] that affect HIV cell entry and replication. Moreover, R5 and X4 HIV-1 may be affected in different ways [82], [83], raising the possibility that coinfecting microbes may play an important role in the switch from R5 to X4 dominance [11].

Nevertheless, the molecular mechanisms of microbial interactions with host cells and especially their effects on HIV-1 infection remain largely unknown. During the last few years it has become evident that the first contact of an invading microbe with the host cell involves engagement of TLRs and other PRRs with microbial molecules inducing an innate immune response.

Here, we tested whether engagement of TLRs by invading microorganisms affects HIV-1 replication in human lymphoid tissues. Because the effects of microbes on host cells are complicated, we used a reductionist approach: instead of whole microbes, we treated human lymphoid tissue *ex vivo* with TLR ligands. We administered various TLR ligands prior to and during HIV-1 infection and monitored HIV-1 replication, cytokine release, expression of selected surface molecules, and cell activation. For these experiments, we used blocks of *ex vivo* human tonsillar tissue in which cytoarchitecture and cellular repertoire, as well as some tissue functions, are preserved. This approach differs from that of previous studies of the effect of TLR engagement on HIV-1 replication *in vitro* that were performed in cell lines, isolated primary cells, human aggregate lymphocyte cultures [47]–[53]. It was shown that at least some events critical for HIV-1 infection (e.g., secretion of cytokines upon infection) significantly differ between *ex vivo* tissue and high density cell aggregates or isolated cells [84].

Tonsils were obtained from patients 2 to 7 years old. At this age, approximately 30% of tonsillar lymphocytes are T cells (CD3^+^) and 70% are B cells (CD19^+^) [85]. Most of the tonsillar lymphocytes express TLR receptors [25], [86]. The majority of other cells present in tonsils, including dendritic cells, NK cells, macrophages, stromal cells, and epithelial cells, are known to express TLRs as well.

Here, we found that TLR ligands affect HIV-1 infection in human lymphoid tissue *ex vivo*. Different TLR ligands differentially affect HIV-1 replication, either up- or downregulating it. We chose TLR5 and TLR9 ligands to investigate the effects of their engagement on HIV-1 infection and the mechanisms for this phenomenon, because of their opposite effects on HIV-1 irrespectively of its coreceptor specificity.

As a TLR5 ligand we used flagellin from *S. typhimurium*. As a TLR9 ligand we used ODN M362 (CpG oligonucleotide type C). There are three types of ODN M362, A, B and C with slightly different structures and eliciting different immune responses [55]–[58]. In the current work we used type C since type C triggers all the pathways triggered by A and B [87]–[90]. Tissue treatment with flagellin led to the increase of both R5 and X4 HIV-1 replication. In contrast, addition of ODN M362 suppressed replication of HIV-1 of both phenotypes. This suppression was partially reversed by a simultaneous addition of a TLR9 antagonist (TTAGGG)4 to R5_SF162_-infected tissues treated with ODN M362.

Treatment of the tonsillar blocks with ODN M362 led to an increase, although transient, of CCL3, CCL4, CCL5, and CXCL12 secretion but not of CXCL10. Also, these cytokines were upregulated when the ODN M362-treated tissues were infected with R5_SF162_. Upregulation of cytokines that bind to HIV coreceptors may be one of the mechanisms mediating ODN M362-induced suppression of HIV-1 replication in the treated tissues.

We found a positive correlation between HIV-1 production and the release of CXCL12 both in ODN M362-treated and in untreated tissues. It seems that it is HIV-1 replication that induces CXCL12 release. In ODN M362-treated cultures, this effect is augmented, i.e., cytokine release in response to HIV-1 replication is increased.

Within the first 60 h post-infection, X4_LAI.04_ virus induced a significantly higher release of CXCL12 from ODN M362-pretreated compared with a ligand-untreated tissue. Since the replication of X4_LAI.04_ virus induces a large release of CXCL12, the effect of ODN M362 on CXCL12 release is masked later in the infection, when significant viral replication occurs. Thus, at that time, most of the observed changes in CXCL12 concentrations reflect changes due to viral replication. To distinguish between contributions of the ligand treatment and X4_LAI.04_ replication to the secretion of CXCL12, we expressed CXCL12 release as a function of HIV-1 p24 release. Our data (see Figure 6) suggest that flagellin suppresses, while ODN M362 enhances, the induction of CXCL12 in response to X4 HIV-1 replication in tonsillar tissue *ex vivo*. The concentrations of the above chemokines detected in the culture media of ODN M362-treated tissue were sufficient to cause suppression of HIV-1 replication [11], [60].

Differences in the production of CXCL10 could also contribute to the modulatory effect of chemokines on HIV-1 replication, since CXCL10 is a potent attractant of activated CD4 T cells [91], the major HIV-1 targets. The increase of this chemokine in HIV-1–infected tissues caused by flagellin and its decrease caused by ODN M362 may be related to their stimulatory or suppressive effects on HIV-1 replication in human lymphoid tissue *ex vivo*.

In general, upregulation of cytokines that bind to HIV coreceptors may be one of the mechanisms mediating ODN M362-induced suppression of HIV-1 replication in the treated tissues.

Cell activation by flagellin, as evidenced by the increase in the fractions of CD4-positive T cells expressing CD25, CD38, and CD39 and especially increase in the number of cycling (Ki67^+^) CD4 T cells, also may contribute to flagellin-related upregulation of replication of HIV-1 of both coreceptor tropisms in lymphoid tissue *ex vivo*. Moreover, it has been reported that flagellin engagement of TLR5 leads to HIV-1 reactivation [52].

ODN M362 inhibitory effect on HIV-1 replication may be mediated by soluble factors induced by this TLR ligand. In particular, it was shown that in response to ODN M362, pDCs produce type 1 IFNs [17], which suppress HIV-1 replication via death of infected cell [92], production of tetherin [93], and/or increased expression of APOBEC3G [94]. In our experiments, we did not detect type 1 IFNs in the culture supernatants of the lymphoid tissue ex vivo although, we readily detected it in supernatants from the tissues spiked with various concentrations of type 1 IFNs. It is nevertheless possible that type 1 IFN is quickly consumed by the neighboring cells without being released into the media in detectable quantities.

Neither of the tested TLR agonists induced a statistically significant loss of CD3^+^CD4^+^CD8^−^ cells such as that reported for TLR ligand-treated PBMC cultures [51], suggesting that different regulations occur in tissues and in isolated cells. Moreover, in tissues TLRs of many cell types can be affected and the final effect of TLR ligands on HIV-1-infected tissues and HIV infection itself could be a superimposition of the responses of various tissue cells that are not necessarily direct HIV-1 targets [16].

In conclusion, we showed that TLR5 and TLR9 ligands affect HIV-1 replication in opposite ways. These ligands changed the cytokine spectra, the cellular activation status, and the expression of cellular receptors in agreement with their up- or downregulation of HIV-1. It is important to keep in mind that although the compounds we and others are using are called TLR ligands, it is possible that they act not only through the TLRs [17]. Nevertheless, TLR seems to be the main pathway triggered by these compounds, and similar pathways may be triggered by actual microbes in infected tissues. Thus, we think that the model described in our paper seems to reflect adequately the mechanism of HIV interaction with other microbes in coinfected tissues. Our results suggest that interactions between coinfecting microbes and HIV-1 can be mediated by PRRs, which trigger the innate immunity response. A particular PRR engaged by a microbe may account for various microbial effects on HIV-1 replication in coinfected tissues and eventually affect the course of HIV-1 disease. Use of engagement of different PRRs by microbial molecules or their mimetics could become a new strategy for HIV therapy or prevention.

## Materials and Methods

### Ethics statement

This study was conducted according to the principles expressed in the Declaration of Helsinki. The study was approved by the Human Subjects Institutional Review Board (IRB) of Children's National Medical Center (Protocol #3204) and by the Office of Human Subjects Research of NIH (OHSR #2264). Since anonymous tissue samples were used, no written informed consent was required.

### Tissue cultures

Anonymous samples of human tonsillar tissues were obtained from patients undergoing routine tonsillectomy at the Children's National Medical Center (Washington, DC). Tissues were dissected into 8- to 27-mm^3^ blocks and placed onto collagen sponge gels in culture medium at the air-liquid interface, as described earlier [95]–[98]. Tissue blocks were cultured in RPMI 1640 (GIBCO BRL, Grand Island, NY) medium containing 15% heat-inactivated fetal calf serum (FCS; Gemini Bio-Products, Woodland, CA), nonessential amino acids (1 mM), sodium pyruvate (1 mM), amphotericin B (2.5 µg/mL; GIBCO BRL), and gentamicin (50 µg/mL; Quality Control, Inc., Rockville, MD). The culture medium was supplemented with Timentin (GlaxoSmithKline, Research Triangle Park, NC) for the first 24 h and then changed regularly. For each condition of infection, 27 blocks were prepared (9 blocks/well/3 ml of complete medium).

### HIV-1 infections

Tissue blocks were infected by application of 5–7.5 µl of HIV stock (0.5–1.0 ng of p24) on top of each tissue block as described previously [11], [98]. X4 isolate LAI.04 (X4_LAI.04_) and R5 isolate SF162 (R5_SF162_) were obtained from the Rush University Virology Quality Assurance Laboratory (Chicago, IL). HIV-1 replication was monitored from measurements of p24 in the culture medium with the Alliance HIV-1 p24 ELISA (PerkinElmer Life Sciences, Inc., Boston, MA).

### TLR ligand treatment

On the second day after cutting, tissue blocks were treated with ligands either by complete replacement of tissue culture media with medium containing TLR ligand (ligands for TLR2, 3, 4, and 7) or by preincubation of the tissue blocks in culture medium containing 250 µl of TLR ligand per 18 blocks for 2 h at 37°C followed by a transfer of the blocks onto collagen sponge gels and subsequent incubation in CO_2_ incubator overnight (ligands for TLR5, 8, and 9). All TLR ligands were obtained from InvivoGen (Chicago, IL) and used at concentrations recommended by the manufacturer as follows: TLR2 agonist LPS from *Porphyromonas gingivalis* (3 µg/ml), TLR3 agonist poly(I:C) (25 µg/ml), TLR4 agonist LPS from *E. coli* K12 (5 µg/ml), TLR5 agonist flagellin (*S. typhimurium*) (5 µg/ml), TLR7 agonist loxoribine (1 mM), TLR8 agonist ssRNA (ssRNA40/LyoVec) (10 µg/ml), and TLR9 agonist CpG oligonucleotide type C (ODN M362) (5 µM). TLR9 antagonist (TTAGGG)4 was used at 5-fold excess (25 µM) over the TLR9 agonist ODN M362. Ligands were kept in culture medium during the whole course of the experiment (ligands for TLR2, 3, 4, and 7) or reapplied in 10-µl quantities on the top of every tissue block after each medium change (ligands for TLR5, 8, and 9). In control cultures, tissue blocks were treated with culture medium only.

### Cytokine measurements

The levels of CCL3, CCL4, CCL5, CXCL10, and CXCL12 were measured in culture supernatants from TLR ligand and/or HIV-1-infected tissues with a multiplexed fluorescent microsphere immunoassay using the Luminex 100 system (Luminex Corporation, Austin, TX). Cytokines, capture antibodies, and biotinylated detection antibodies were obtained from R&D System (Minneapolis, MN). We coupled 100 µg of cytokine capture antibodies covalently to 12.10^6^ carboxylated microspheres using sulfo-NHS and EDC according to the Luminex standard protocol. We mixed 1,200 coupled microspheres from each set with 50 µl of standards or culture medium and incubated them overnight at 4°C in a multiscreen filter plate (Millipore corporation, Billerica, MA). After three washes by vacuum aspiration, 50 µl of biotinylated polyclonal anti-cytokine antibodies were added to each well and incubated for one hour at 37°C. Following three additional washes, the bound cytokines were detected by the addition of 50 µl of a 16-µg/ml solution of streptavidin-phycoerytrin (Molecular Probes, Carlsbad, CA). Data were collected and analyzed with Bioplex Manager v3.0 software (Bio-Rad, Hercules, CA) using a 5-parameter fitting algorithm.

### Flow cytometry

Flow cytometry of cells isolated from tissue blocks and stained for cell surface markers with specific antibodies was performed as described earlier [99]. Briefly, cell that migrated from the tissue blocks were collected from the collagen sponge gels and tissue culture medium. Additionally, single-cell suspensions were prepared from tonsillar tissue blocks by digestion with Collagenase IV (GIBCO BRL) at 2.5 mg/ml in RPMI supplemented with 5% FCS for 30 min. Cell suspensions were passed through a 40 µm nylon mesh (Falcon) and stained with labeled monoclonal antibodies coupled to fluorochromes. Staining was performed in three groups. The first group was stained with annexin V labeled with PE and with combinations of the following labeled monoclonal antibodies: anti-CD3-APC-Cy5.5, anti-CD4-PE-Alexa 610, anti-CD8-PacificBlue, anti-CD38-PE-Cy7, anti-CD45RO-APC, and anti-CD69-PE-Cy5.5. The second group was stained with annexin V labeled with PE and with combinations of the following labeled monoclonal antibodies: anti-CD3-APC-Cy5.5, anti-CD4-PE-Alexa 610, anti-CD8-PacificBlue, anti-CD14-PE-Cy5.5, anti-CD19-Tricolor, anti-CCR5-APC-Cy7, and anti-CXCR4-APC. A third group was stained with combinations of the following labeled monoclonal antibodies: anti-CD3-NC650, anti-CD4-NC605, anti-CD8-eFluor 450, anti-CD45-AlexaFluor 780, anti-CD38-AlexaFluor 700, anti-CD39-APC, anti-CD25-Cy7-PE, anti-HLA DR-Cy5.5-PE, anti CD154-Cy5-PE, anti-CD69-PE. Antibodies used in the first and second staining group were purchased as follows: Antibodies to CD3, CD4, CD8, CD14, CD19, CD45RO, and CD69 were products of Invitrogen/Caltag Laboratories, Burlingame, CA. Annexin V and antibodies to CD38, CD184 (CXCR4), and CD195 (CCR5) were purchased from Becton-Dickinson (Pharmingen, San Jose, CA). Antibodies used in the third staining group were purchased from eBioscience (antibodies to CD3, CD4, CD8, CD45, CD39, CD25), from Biolegend (antibodies to CD38, CD154, CD69), and from Invitrogen (antibodies to HLA DR). After staining, cells were washed and fixed in PBS containing 2% formaldehyde. Data were acquired with an LSRII flow cytometer equipped with 355-, 407-, 488-, 532-, and 638-nm LASER lines using DIVA 4.1.2 software (Becton Dickenson). Data were compensated and analyzed with FlowJo version 9 (Treestar, Ashland, OR, USA). Cell depletion was quantified by addition of True Count beads (Invitrogen/Caltag Laboratories) to each tube prior to acquisition as a volumetric control and by normalization of the number of cells by tissue-block weight.

### Expression of data and statistical analysis

Viral replication is expressed as the concentration of p24 in culture medium pooled from 27 blocks (three wells) accumulated during the particular time period. As reported earlier [95]–[98], there was a substantial donor-to-donor variability in HIV replication. To be able to pool and compare the results obtained from tissues of different donors, we adjusted the results using the data from matched control blocks as the basis of normalization. Cytokine secretions are expressed as fold increase (the ratio of cytokine production in infected or coinfected tissue to cytokine production in control cultures) or as actual concentrations. The data obtained in treated tissues from different donors were pooled after being normalized and expressed as percent of the data from matched uninfected untreated control blocks at the corresponding time points. Statistical significance was assessed with nonparametric tests such as the Wilcoxon signed-rank test was used for data that did not pass the normality test. All the hypothesis tests were two-tailed, and a *p* value of ≤0.05 was considered statistically significant.
